# The Impact of Age Difference on the Efficacy and Safety of COVID-19 Vaccines: A Systematic Review and Meta-Analysis

**DOI:** 10.3389/fimmu.2021.758294

**Published:** 2021-12-06

**Authors:** Jiting Wang, Yue Tong, Duo Li, Jun Li, Yaling Li

**Affiliations:** ^1^ Department of Pharmacy, The Affiliated Hospital of Southwest Medical University, Luzhou, China; ^2^ Department of Pharmacy, Southwest Medical University, Luzhou, China; ^3^ Department of Pharmaceutical Chemistry, Southwest Medical University, Luzhou, China; ^4^ Department of Respiration, The Affiliated Hospital of Southwest Medical University, Luzhou, China; ^5^ Department of Traditional Chinese Medicine, The Affiliated Hospital of Southwest Medical University, Luzhou, China

**Keywords:** efficacy and safety, COVID-19 vaccines, age, randomized-controlled trials (RCT), double-blind, meta-analysis

## Abstract

**Objective:**

This meta-analysis compared the efficacy and safety of five kinds of COVID-19 vaccines in different age groups (young adults and older adults), aiming to analyze the difference of adverse events (AEs) rate and virus geometric mean titer (GMT) values between young and older people, in order to find a specific trend, and explore the causes of this trend through meta-analysis.

**Method:**

Meta-analysis was used to analyze the five eligible articles. The modified Jadad scoring scale was used to evaluate the quality of eligible literature with a scoring system of 1 to 7. The primary endpoint of the effectiveness index was GMT. The primary endpoints of the safety index were the incidence of local AEs and systemic AEs. Stata 12.0 software was used for meta-analysis. Revman 5.0 software was used to map the risk of publication bias, and Egger’s test was used to analyze publication bias.

**Results:**

The GMT values of young adults were higher than older adults (SMD = 1.40, 95% CI (0.79, 2.02), P<0.01). There was a higher incidence of local and systemic AEs in young people than in the elderly (OR = 1.10, 95% CI (1.08, 1.12), P<0.01; OR = 1.18, 95% CI (1.14, 1.22), P<0.01).

**Conclusion:**

The immune effect of young people after being vaccinated with COVID-19 vaccines was better than that of the elderly, but the safety was worse than that of old people, the most common AEs were fever, rash, and local muscle pain, which were tolerable for young people. As the AEs of the elderly were lower, they can also be vaccinated safely; the reason for the low level of GMT in the elderly was related to Immunosenescence. The vaccine tolerance of people of different ages needs to be studied continuously.

## Introduction

It has been almost 2 years since the outbreak of coronavirus pneumonia (COVID-19). According to the data of the World Health Organization (WHO), as of June 28th, 2021, central European time, the total number of confirmed cases of COVID-19 in the world were about 182 million, the total number of deaths was about 3.9 million (1), and these numbers are still increasing at an alarming rate every day. The global situation is still severe, meanwhile, the epidemic of COVID-19 has become the primary health threat to all humankind, and politics, economy, and culture worldwide have also been greatly impacted (2). Severe Acute Respiratory Syndrome Coronavirus 2 (SARS-CoV-2) that leads to COVID-19 is a β coronavirus with RNA as a genetic substance, entering the cell by protein binding angiotensin transformase 2 (3). SARS-CoV-2 appears to spread faster than other coronaviruses, leading to an urgent need for COVID-19 vaccines (4). People aged 60 years old or older and those with existing respiratory or cardiovascular diseases are at high risk of serious disease and death if they are infected with SARS cov-2, so the elderly need to be vaccinated (5).

According to different targets and technologies, vaccines can be divided into the following categories: inactivated vaccines, recombined spike protein vaccines, viral vector vaccines, RNA vaccines, live attenuated vaccines, and virus-like particle vaccines (6, 7). The number of vaccinations worldwide at date is about 2 billion, and it took only a little more than 6 months to achieve this milestone (8).However, this was then followed by all kinds of concerns about the vaccines; for example, will the elderly face more serious AEs or have a higher incidence of AEs? Will the amount of antibodies produced by the elderly be less than that of the young? As the world continues to approve COVID-19 vaccines, the frail elderly are center stage as most of the excess deaths occur in the elderly group (9). Residents in care homes and older people with co-morbidities are likely to be among the first to be vaccinated (10). Meanwhile, a lot of misinformation about vaccines spread in social media and other places makes this task a major public health challenge (11). So we need to battle misinformation with the aggressive dissemination of accurate information about the realities of COVID-19 and the risks and benefits of vaccination.

The safety and effectiveness of the vaccines are investigated by randomized controlled trials, and the information of participants in the authorized trials including age, race, and racial background was provided (12). According to the WHO draft of COVID-19 candidate vaccines, 42 candidate vaccines were evaluated clinically and 151 candidate vaccines were evaluated preclinically (13). Some of the candidate vaccines showed safety and immunogenicity in clinical trials, which laid a foundation for studying the age differences in the effectiveness and safety of vaccines. Although the results of the phase 3 clinical trials of several vaccines have been published, there are few studies about the effect of age difference on the safety and efficacy of vaccines. Thus, we included 5 qualified literatures and used the method of meta-analysis to explore the effect of age difference on the efficacy and safety of COVID-19 vaccines, so as to provide a reference for people who still have concerns about vaccination.

## Materials and Methods

### Data Sources and Searches

Embase, MEDLINE/Ovid, Epistemonikos, and Cochrane were searched from inception until April 17, 2021. In addition, we also manually searched for articles that met the criteria. The search terms contained efficacy, safety, COVID-19 vaccines, age, randomized-controlled trials, double-blind, and meta-analysis. The questions for this systematic review were developed using the PICO (population, intervention, comparator, outcome) criteria:

-Population: total 13,209 young participants, 9703 older participants; young adults aged 16-59; older adults aged ≥55.
-Intervention: young adults group and older adults group were injected with SCB-2019, BBIBP-CorV, BNT162b1, BNT162b2, and Ad26.COV2.S vaccines. And two groups received SCB-2019, BNT162b1, BNT162b2, Ad26.COV2.S with two doses (first dose/second dose, low dose/high dose).
-Comparator: experimental group (young adults) vs control group (older adults).
-Outcome: efficacy index was GMT at days 29, 35, 36; safety indexes were the incidence of local AEs and systemic AEs after the first dose, second dose, low dose, and high dose.

### Study Selection

We included randomized-controlled trials (RCTs) that evaluated the efficacy and safety of COVID-19 vaccines in young adults and older adults. The criteria are as follows:

Inclusion criteria:

- RCTs that included young people aged 16 to 59 years and older people aged ≥55 years with different types of COVID-19 vaccines.
- RCTs that explored the efficacy and safety of different types of COVID-19 vaccines.
- Both single dose and double dose COVID-19 vaccines were included.

Exclusion criteria:

- Systematic reviews without meta-analyses.
- Animal or *in vitro* models.
- No peer-reviewed article.
- Conference abstracts.
- Unable to extract valid data.

### Data Screening and Data Extraction

Duplicates exclusion was implemented by two independent reviewers. If there was no consensus, the conflict was solved by a third reviewer. Two independent investigators extracted the following information from each article: (I) publication time; (II) corresponding author and first author; (III) PMID/DOI; (IV) population and main condition of patients in RCTs; (V) name of COVID-19 vaccines; (VI) Single-dose or double dose of COVID-19 vaccine; (VII) number of included studies and the total number of people included in the meta-analysis; (IX) number of cases for each age group; (VIII) study design of included primary studies (RCT); (X) age range of young group and older group; (XI) primary effectiveness index; (XII) primary safety index.

### Risk of Bias and Quality Assessment

The modified Jadad Scoring Scale (14) was used to evaluate the quality of eligible literature with a scoring system of 1 to 7. Random sequence generation, blind method, randomized allocation concealment, and patient withdrawal were evaluated. Jadad scores of 4 to 7 were considered high-quality literature, and 1 to 3 were low-quality literature. The Cochrane risk of bias assessment tool was used to assess the methodological quality of individual studies based on the following aspects: random sequence generation, allocation concealment, blinding of participants and personnel, blinding of outcome and assessment, incomplete outcome data, selective reporting, and other bias. Each item was answered with a high, low, or unclear risk of bias, and disagreements were resolved through an open discussion or a third reviewer. The general chart of bias risk was made by Revman 5.0 software.

### Statistical Analysis

Stata 12.0 software was used for meta-analysis. The binary variables were expressed by odds ratio (OR) and 95% confidence interval (CI); the continuous variables were represented by standardized mean difference (SMD) and 95% CI. If there was no statistical heterogeneity among the studies (P > 0.1, I^2^ < 50%), the fixed effects model was used for analysis; otherwise, the random effect model was used for analysis. Revman 5.0 software was used to map the risk of publication bias, and Egger’s test was used to analyze publication bias. P < 0.05 was statistically significant.

## Results

### Systematic Literature Search

**Figure 1:**
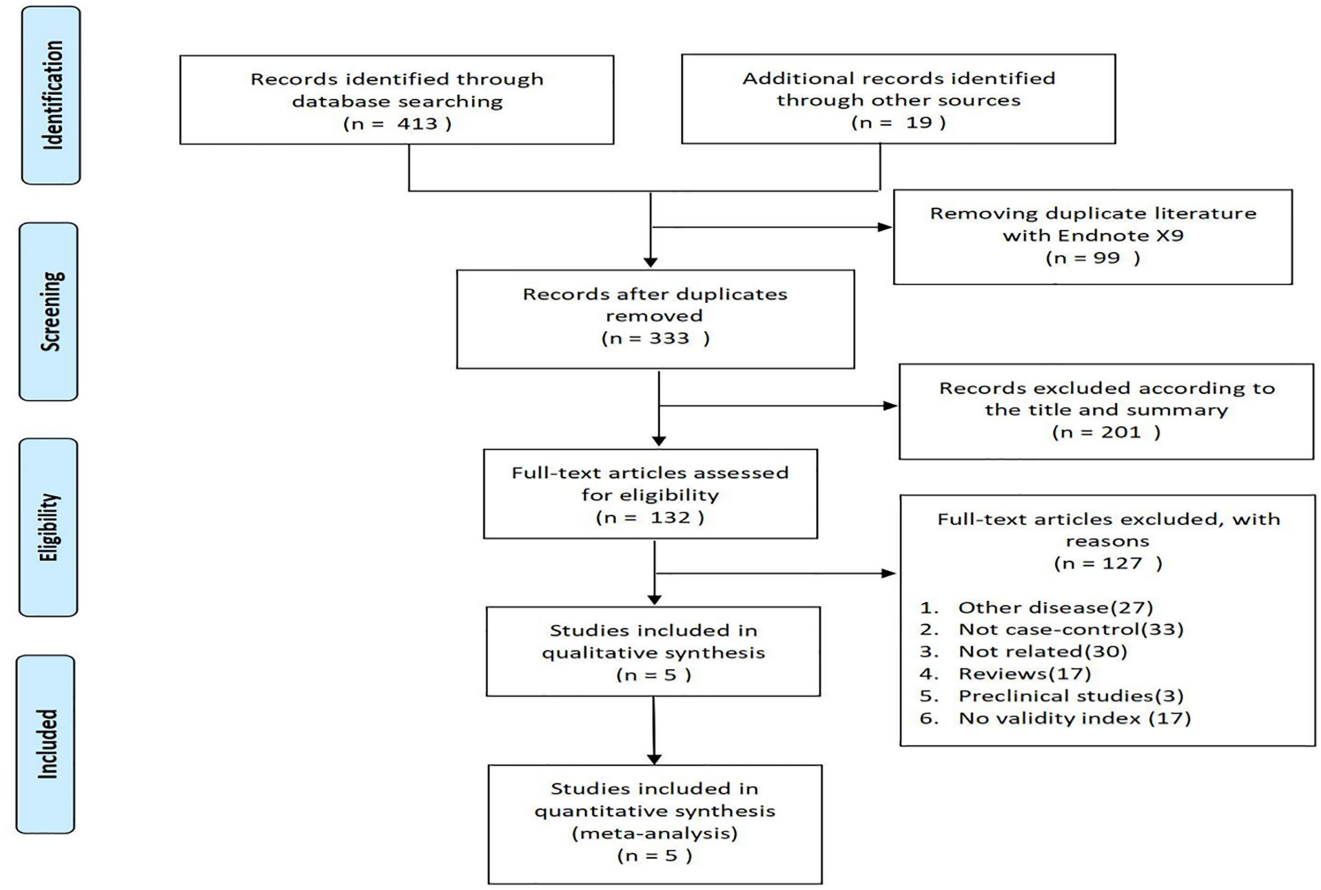
Flowchart of studies evaluating qualified research through selection process.

The flowchart of PRISMA (preferred reporting items for systematic review and meta-analysis) was shown in **Figure 1**. A total of 432 potentially relevant articles were included in the combined electronic and paper reference search. After preliminary screening, 300 publications were excluded according to the title and abstract. After detailed reading and evaluation of the full text, another 127 articles were excluded because they had no valid data to extract, or they were animal or basic studies, or they were reviews without meta-analysis, or they were conference abstracts. Overall, 5 RCTs were included, involving 22,552 young and older adults.

### Study Characteristics and Quality Assessment

The details of the COVID-19 vaccines, baseline characteristics of the populations, age range, study period, dosage, efficacy Index, and safety index of five eligible trials are shown in **Table 1**. The improved Jadad Scale (14) was used to evaluate its quality. The Jadad scores were between 6 and 7 and were all high-quality documents (**Table 1**).

**Table 1:** Characteristics of included studies and Jadad scores.

Study	Jadad	vaccine	N (young/old)	Age range (young/old)	Country	Study Types	Administration	Efficacyindex and Safety index
Richmond et al. (15)	7	SCB-2019	90/60	18-54/55-75	Western Australia	RCT, double-blind, one center	First dose + second dose	Local AEs, systemic AEs, GMT
Xia et al. (16)	7	BBIBP-CorV	72/72	18-59/≥60	China	RCT, double-blind, one center	Single dose	Local AEs, systemic AEs
Walsh et al. (17)	6	BNT162b1 and BNT162b2	144/144 and 144/144	18-55/65-85	United States	RCT, observer-blinded, one center	First dose + second dose	Local AEs, systemic AEs, GMT
Sadoff et al. (18)	7	Ad26.COV2.S	162/161	18-55/≥65	Belgium and United States	RCT, double-blinded, multi-center	Low dose + high dose	Local AEs, systemic AEs, GMT
Skowronski and De Serres (19)	6	BNT162b2	12597/9122	16-55/≥55	United States, Argentina, Brazil, South Africa, Germany, Turkey,	RCT, observer-blinded, multi-center	First dose + second dose	Local AEs, systemic AEs

### Efficacy Analysis

**Figure 2:**
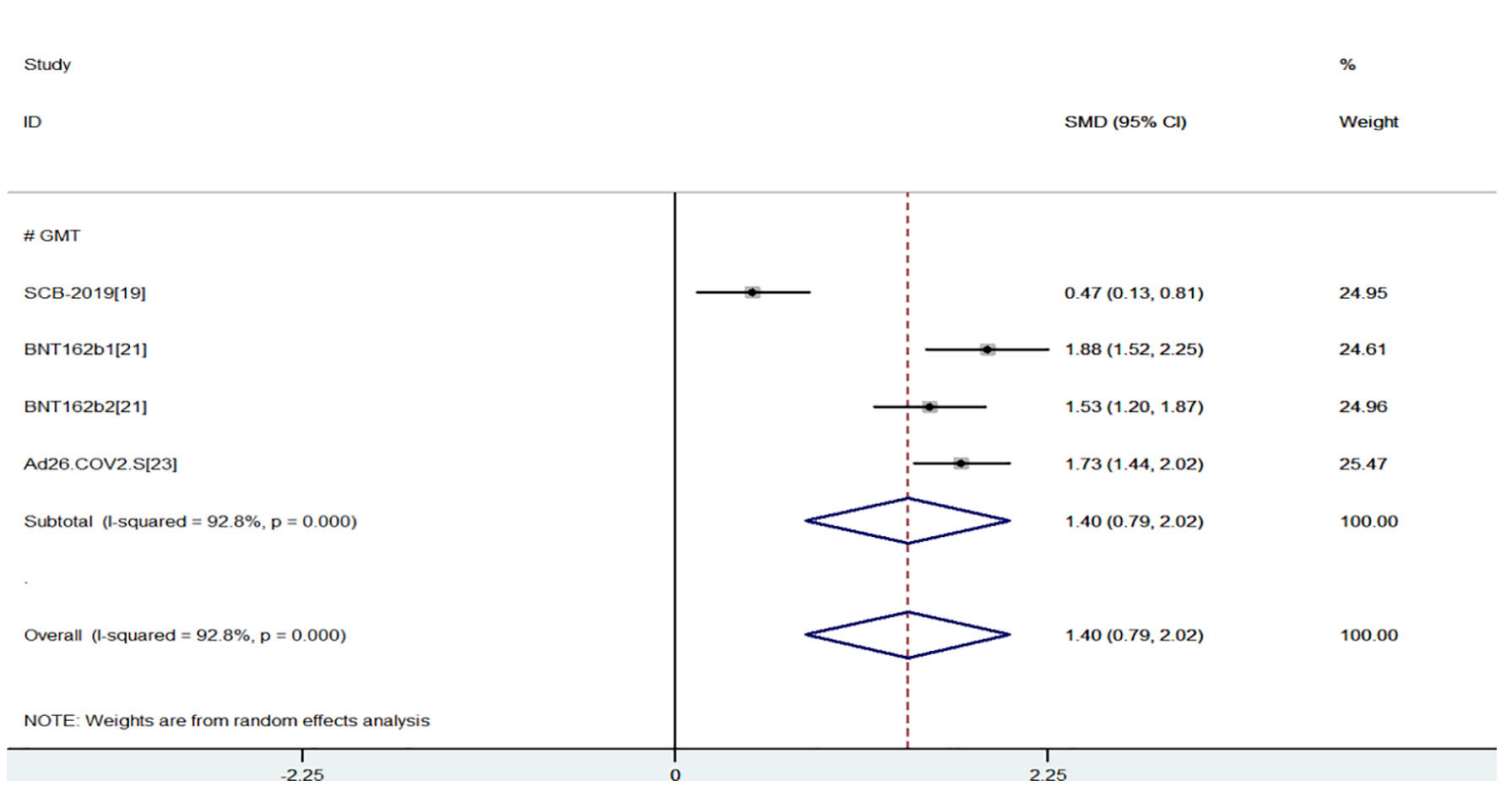
Meta-analysis of GMT between the experimental group (young adults) vs control group (older adults).

**Figure 3:**
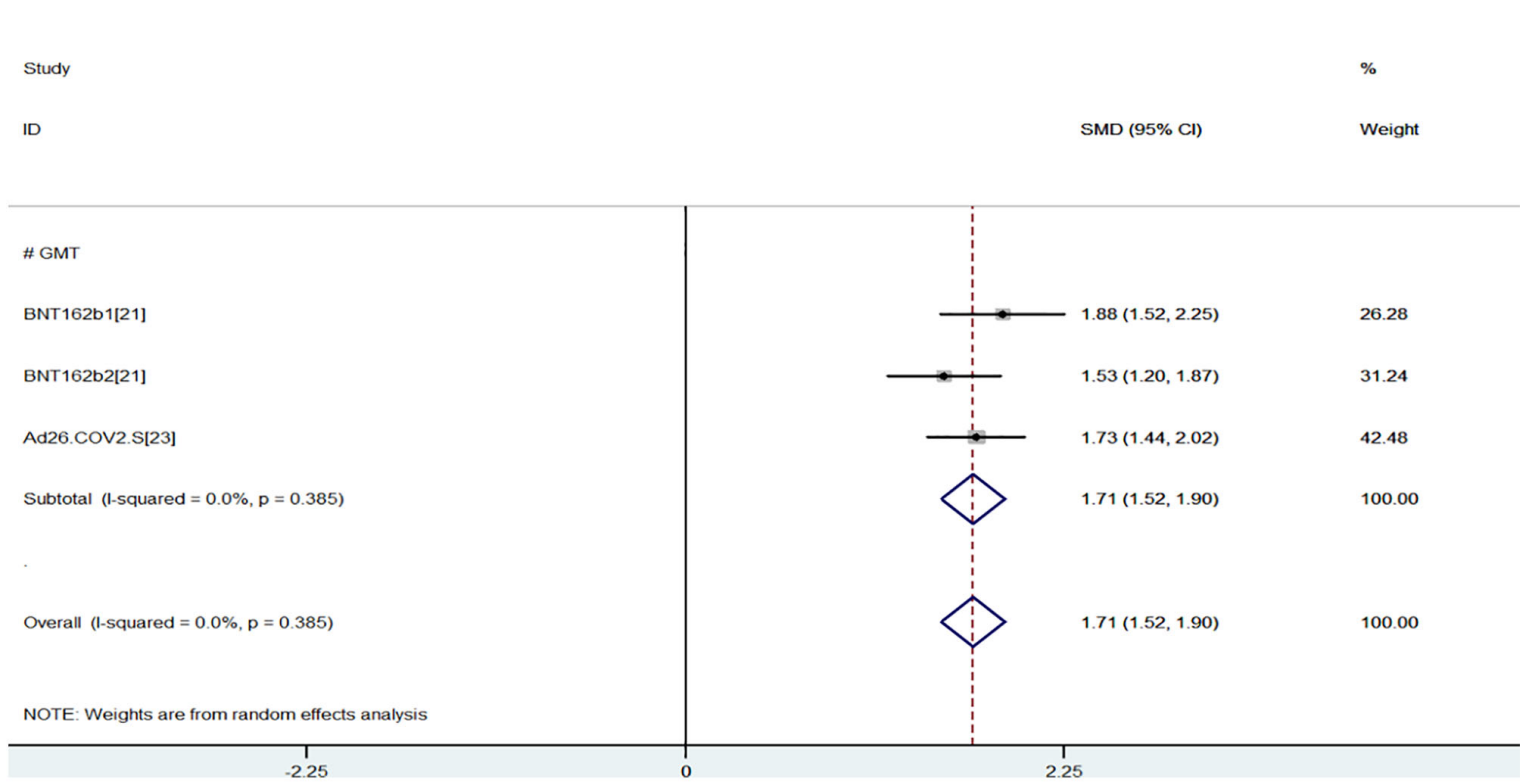
Meta-analysis of GMT between the experimental group (young adults) vs control group (older adults).

The efficacy index was GMT values of neutralizing antibody measured by live virus neutralization test at 29, 35, and 36 days after last vaccination. The greater the GMT, the stronger the immunogenicity. The results of the meta-analysis of effectiveness indicator were as follows, and the random-effects model was used for analysis: When compared with the young group, the virus GMT values of the older group were lower (SMD = 1.40, 95% CI (0.79, 2.02), P<0.01), suggesting that young group had better immunogenicity and had more advantages in efficacy index (15, 17, 18) (**Figure 2**). However, due to the high heterogeneity (93%), sensitivity analysis found that the first study (15) had the lowest overlapping rate of confidence intervals, and the heterogeneity (0.0%) returned to normal after excluding the first study (15) (SMD = 1.71, 95% CI (1,52, 1.90), P<0.01) (**Figure 3**).

### Safety Analysis

**Figure 4:**
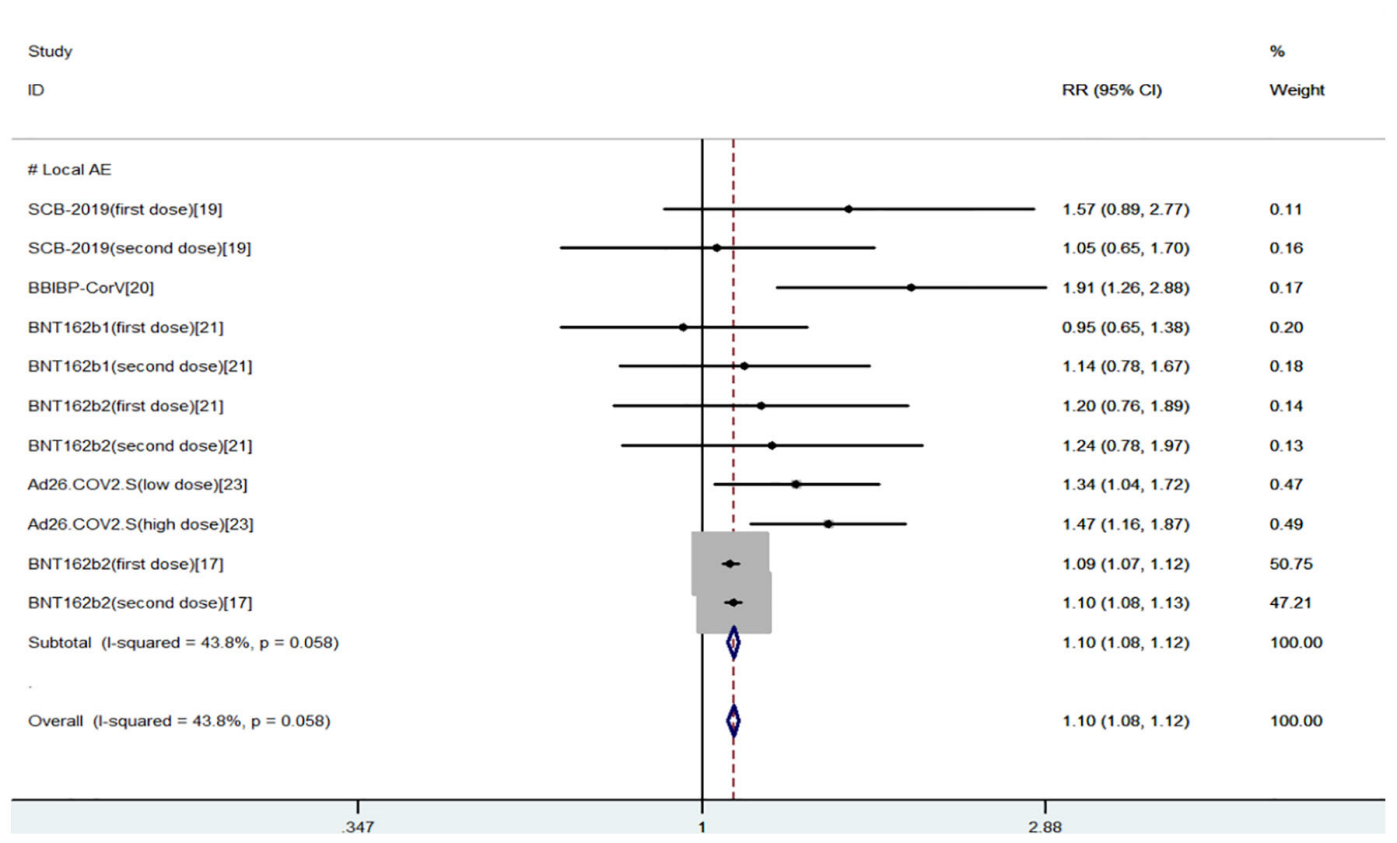
Meta-analysis of local AEs between the experimental group (young adults) vs control group (older adults).

**Figure 5:**
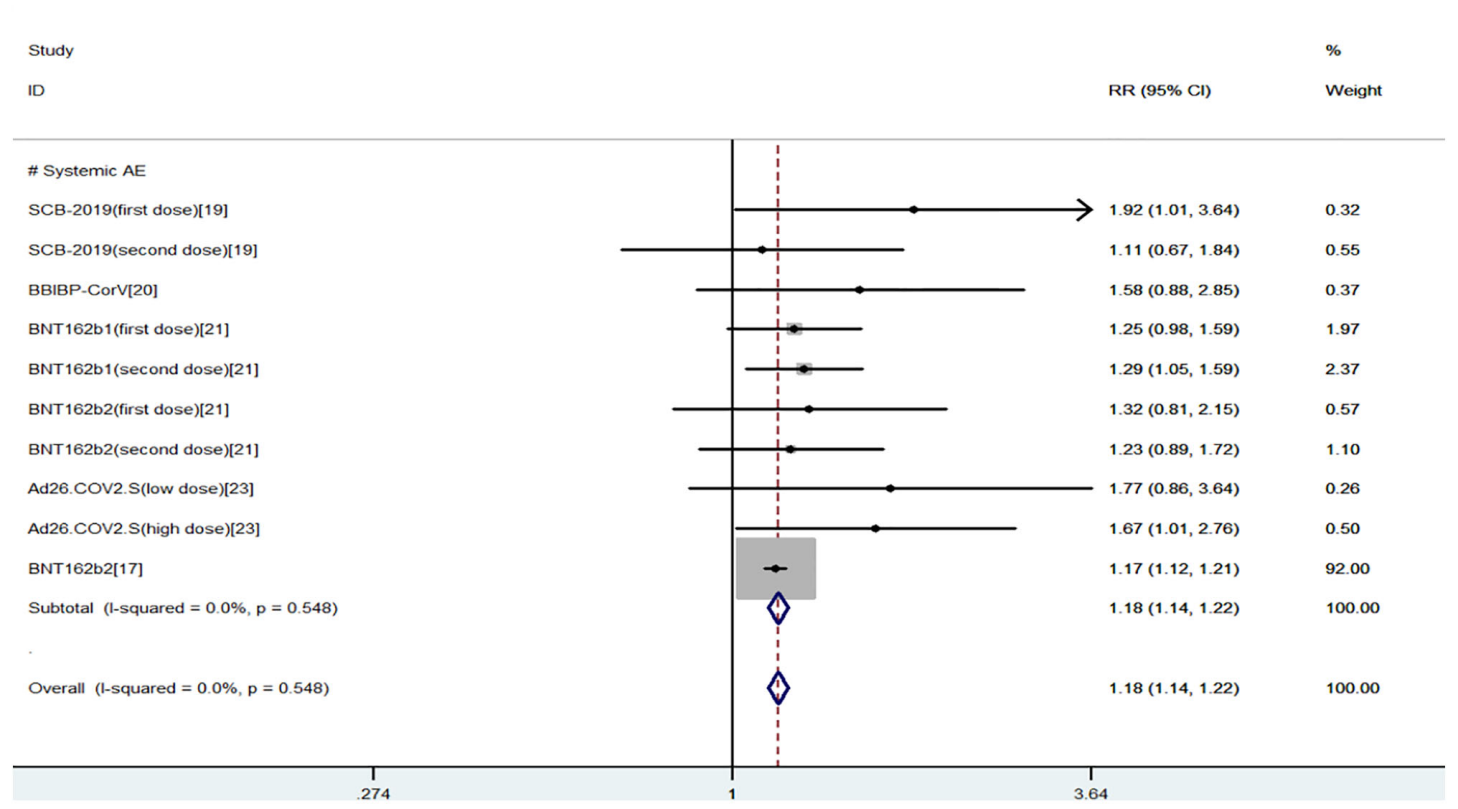
Meta-analysis of systemic AEs between the experimental group (young adults) vs control group (older adults).

The safety indexes were local AEs and systemic AEs. Common local AEs included injection site pain, itching, redness, swelling, and rash. Common systemic AEs included fever, fatigue, nausea, headache, cough, diarrhea, and muscle pain (20, 21). The results of the meta-analysis of safety indicators were as follows, and the fixed effects model was used for analysis: We found that there was a higher incidence of local and systemic AEs in young people than in the elderly (OR = 1.10, 95% CI (1.08, 1.12), P<0.01; OR = 1.18, 95% CI (1.14, 1.22), P<0.01), the heterogeneity were within the required range, suggesting that COVID-19 vaccines had more advantages in safety index for the elderly (15–19) (**Figures 4**, **5**).

### Heterogeneity and Risk of Bias

**Figure 6:**
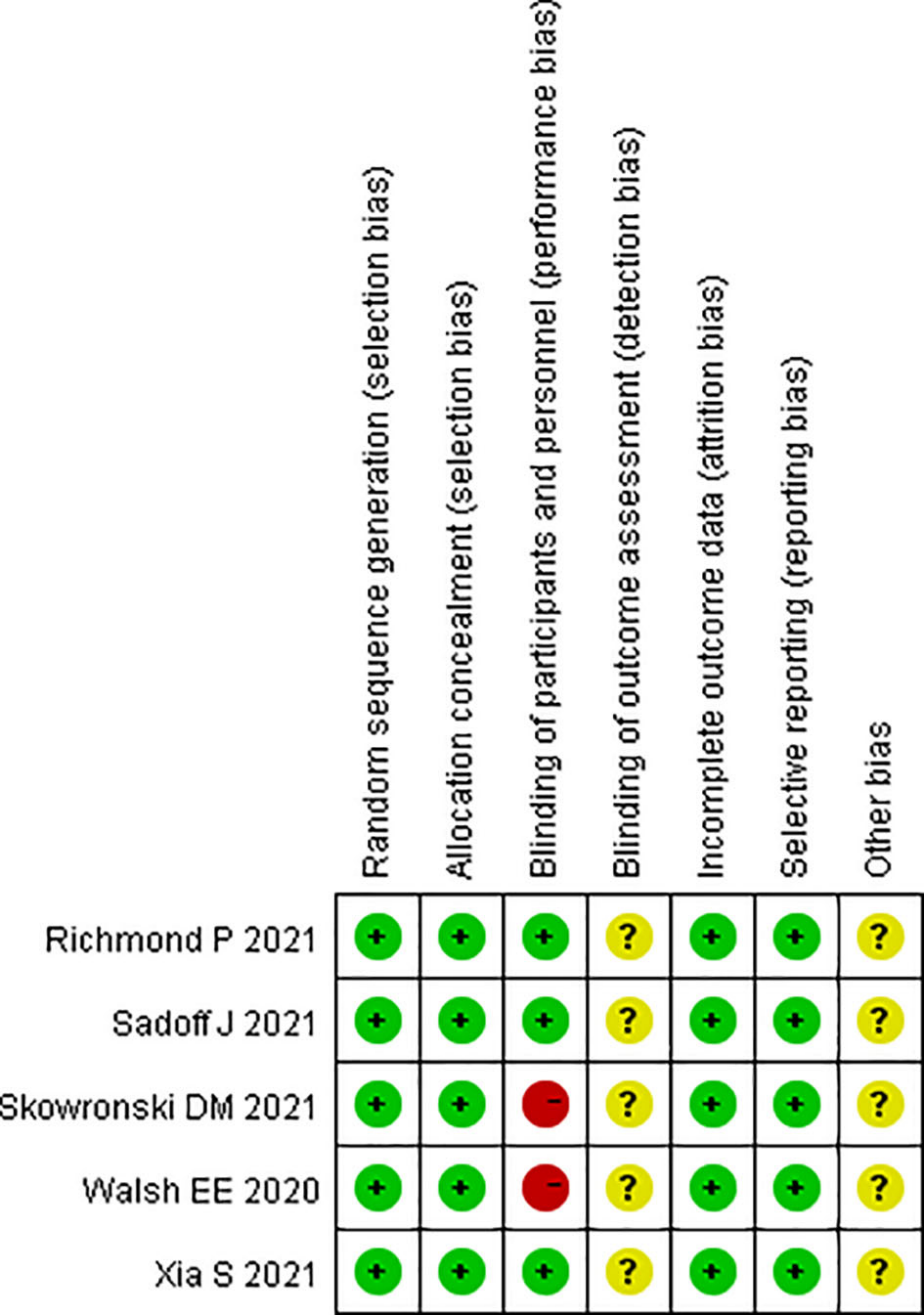
Summary chart of bias risk.

The fixed-effect model was used to analyze the efficacy and safety indicators, and the heterogeneity generally met the requirements after sensitivity analysis. Local AEs and systemic AEs were used as indicators (15–19) for publication bias analysis. Egger’s test was used for the calculation of publication bias analysis. The result of Egger’s test (P = 0.41 > 0.05) indicated that there was less possibility of publication bias. The RevMan 5.0 software was used to assess the methodological quality of individual studies based on the following aspects: random sequence generation, allocation concealment, blinding of participants and personnel, blinding of outcome and assessment, incomplete outcome data, selective reporting, and other bias. Each item was answered with a high, low, or unclear risk of bias, and disagreements were resolved through an open discussion or a third reviewer (**Figure 6**).

## Discussion

COVID-19 is still prominent around the world. According to the current international epidemic situation, the epidemic will continue for a long time into the future (22). Even in countries where the epidemic is under control, the outbreak of COVID-19 is also likely to be more prevalent due to the introduction of new infectious sources at any time (8). Therefore, we must vaccinate more so that more people can gain immunity because vaccination is the most effective way to prevent and control COVID-19 at present (23). If there are spare vaccines left over in refrigerators and vials, all efforts should be made to inoculate them into the human body, so they can do their work (24). In this meta-analysis, five related literatures were included, which were SCB-2019, BBIBP-CorV, BNT162b1, BNT162b2, and Ad26.COV2.S. RCTs of the above five vaccines had a very comprehensive dose study (15–19), including single-dose and double-dose studies, low-dose and high-dose studies. Exploring the differences of effectiveness and safety of the above five vaccines in different age groups (young and old) can help people correctly understand the advantages and disadvantages of vaccines for different age groups, and carry out vaccination with an objective and scientific attitude, which has good practical significance.

The results of the meta-analysis of the effectiveness indicator showed that when compared with the older group, the virus GMT values of the young group were higher, suggesting that the young group had better immunogenicity, most of the AEs are fever, rash, fatigue, and local pain, which are tolerable for young people (15, 17, 18); the results of the meta-analysis of safety indicators showed that there was a lower incidence of local and systemic AEs in older people than in the young, suggesting that COVID-19 vaccines are not only effective but also had more advantages in safety index for the elderly (15–19), which means the elderly can dispel their concerns about the safety of vaccines. Meanwhile, the reason for the lower GMT values in the elderly is Immunosenescence, which is a new concept that reflects the age-associated restructuring changes of innate and adaptive immune functions (25). The changes of immune organs in the elderly are most obvious in the thymus, the activity of thymocytes and thymic epithelial cells in the elderly are reduced, the immune response substances are reduced, and therefore the immune function is decreased (26, 27). In conclusion, both the elderly and young people are encouraged to take the COVID-19 vaccines as soon as possible.

Most of the previous single RCT about COVID-19 included the elderly and young people, but these studies were not collected for systematic meta-analysis. So this study creatively used the meta-analysis method to integrate all relevant literature to study the differences in safety and efficacy between the elderly and young after COVID-19 vaccination, in order to provide a reference for the elderly and young with vaccination concerns. The advantages of this meta-analysis are that the included articles are of high quality, the design of RCTs is scientific and reasonable, and it also has good practical significance to provide an evidence-based reference for those, elderly and young, who still have concerns about vaccination. However, this meta-analysis still had some limitations. First, the sample size of RCTs of efficacy and safety of COVID-19 vaccines in different age were small because of the lack of existing research. Second, two included studies (17, 19) are observer-blind rather than double-blind, which may be the reason for selection bias. Thirdly, the meta-analysis of GMT showed great heterogeneity, and after sensitivity analysis, the first study (15) was excluded, then the heterogeneity returned to normal. Therefore, this conclusion needs to be further confirmed by more high-quality, multi-center, and large-sample researches.

## Conclusion

The immune effect on young people after being vaccinated with COVID-19 vaccines was better than that of the elderly, but the safety was worse than that of old people; the most common AEs were fever, rash and local muscle pain, which were tolerable for young people. As the AEs of the elderly were lower, they can also be vaccinated safely, the reason for the low level of GMT in the elderly was related to Immunosenescence. The vaccine tolerance of people of different ages needs to be studied continuously.

## Author Contributions

JW, YT, and YL carried out the conception and design of the article and wrote the paper. JW and YT carried out the implementation and feasibility analysis of the research. JW and DL collected the data. JL carried out the data sorting. JW and YT carried out the statistical processing. JL and DL carried out the analysis and interpretation of the results and the revision of the paper. YL was responsible for the quality control and review of the article and analyzed the overall article. All authors contributed to the article and approved the submitted version.

## Funding

This study was supported by the Sichuan Provincial Department of Education (SCYG2019-04, YF19-Y12).

## Conflict of Interest

The authors declare that the research was conducted in the absence of any commercial or financial relationships that could be construed as a potential conflict of interest.

## Publisher’s Note

All claims expressed in this article are solely those of the authors and do not necessarily represent those of their affiliated organizations, or those of the publisher, the editors and the reviewers. Any product that may be evaluated in this article, or claim that may be made by its manufacturer, is not guaranteed or endorsed by the publisher.
