# Supplementary material for: The Impact of Age Difference on the Efficacy and Safety of COVID-19 Vaccines: A Systematic Review and Meta-Analysis
Source: Front Immunol. 2021 Dec 6;12:758294. doi: 10.3389/fimmu.2021.758294 (PMC8685545; doi:10.3389/fimmu.2021.758294)
Supplement: Supplementary file 1 [file Table_1.pdf]

Status of COVID-19 Vaccines within WHO EUL/PQ evaluation process

	Manufacturer / WHO EUL holder	Name of Vaccine	NRA of Record	Platform	EOI accepted	Pre-submission meeting held	Dossier accepted for review*	Status of assessment**	Decision date***
1.	Pfizer BIONTECH	BNT162b2/COMIRNATY<br>Tozinameran (INN)	EMA	Nucleoside modified mNRA	✓	✓	✓	Finalized	31/12/2020
							✓	Finalized:<br>– Baxter Oncology GmbH Germany (DP)<br><br>Diluent suppliers:<br>– Pfizer Perth, Australia<br>– Fresenius Kabi, USA	30/06/2021<br><br><br>18 June 2021<br><br>18 June 2021
							Additional sites, awaited :<br>• Polymun Scientific, Austria (DP)<br>• Pharmacia & Upjohn, Kalamazoo (DP)<br>• mibe (Dermapharm), Germany (DP)	• Submission awaited<br>• Submission awaited<br>• Submission awaited	• As submiiited<br>• As submiiited<br>• As submiiited
2.	AstraZeneca OXFORD	AZD1222	EMA	Recombinant ChAdOx1 adenoviral vector encoding the Spike protein antigen of the SARS-CoV-2.	✓	✓	✓	Core data finalized	16 April 2021
							Data for Covax sites expected in April 2021 onwards	Finalized: SK-Catalent<br>Wuxi (DS)<br>Chemo Spain<br><br>Other sites	16 April 2021<br>30 April 2021<br>04 June 2021<br><br>As submitted
3.	AstraZeneca OXFORD	AZD1222	MFDS KOREA	Recombinant ChAdOx1 adenoviral vector encoding the Spike protein antigen of the SARS-CoV-2.	✓	✓	✓	Finalized	15 Feb 2021
4.	SERUM INSTITUTE OF INDIA PVT. LTD. Cyrus Poonawalla Group	Covishield (ChAdOx1_nCoV-19)	DCGI	Recombinant ChAdOx1 adenoviral vector encoding the Spike protein antigen of the SARS-CoV-2.	✓	✓	✓	Finalized	15 Feb 2021
5.	Janssen Infectious Diseases & Vaccines	Ad26.COV2.S	EMA	Recombinant, replication-incompetent adenovirus type 26 (Ad26) vectored vaccine encoding the (SARS-CoV-2) Spike (S) protein	✓	✓	✓	Core data finalized (US +NL sites)	12 March 2021
							Additional sites:<br>- Aspen RSA (DP)<br>- Catalent Agnani Italy (DP)<br>- Merck, Durham, UK (DS)<br>- Merck, West Point/PA (DP)	- Finalized<br>- Ongoing<br>- Future submission<br>- Future submission	-25 June 2021<br>- 02 July 2021<br>- As submitted<br>- As submitted
6.	moderna	mRNA-1273	EMA	mNRA-based vaccine encapsulated in lipid nanoparticle (LNP)	✓	✓	✓	Finalized	30 April 2021
							Additional sites, awaited:<br>- ModernaTx. Norwood (DS)<br>- Catalent Indiana, LLC (DP)<br>- Lonza Biologics, Inc. Portsmouth, USA (DS)<br>- Baxter, Bloomington, USA (DP)	- Ongoing<br>- Ongoing<br>- Ongoing<br><br>- Ongoing	- As submitted<br>- As submitted<br>- As submitted<br><br>- As submitted

	Manufacturer / WHO EUL holder	Name of Vaccine	NRA of Record	Platform	EOI accepted	Pre-submission meeting held	Dossier accepted for review*	Status of assessment**	Decision date***
7.	Sinopharm / BIBP[1]	SARS-CoV-2 Vaccine (Vero Cell), Inactivated (InCoV)	NMPA	Inactivated, produced in Vero cells	✓	✓	✓	Finalized	07 May 2021
8.	sinovac	COVID-19 Vaccine (Vero Cell), Inactivated/ Coronavac™	NMPA	Inactivated, produced in Vero cells	✓	✓	✓	Finalized	01 June 2021
9.	THE GAMALEYA NATIONAL CENTER	Sputnik V	Russian NRA	Human Adenovirus Vector-based Covid-19 vaccine	Additional information submitted	Several meetings have been and continue to be held.	"Rolling" submission of clinical and CMC data has started.	Additional data (Non-CLIN, CLIN, CMC) Required. Following up on inspection observations.	Anticipated date will be set once all data is submitted and follow-up of inspection observations completed.
10.	AstraZeneca / OXFORD	AZD1222	Japan MHLW/PMDA	Recombinant ChAdOx1 adenoviral vector encoding the Spike protein antigen of the SARS-CoV-2.	Submission from AZ received on 15 June. MHLW submiited review and GMP reports on 16 June 2021 and June 22.	Several meetings held separately with AZ and MHLW/PMDA	✓	Ongoing	Anticipated date: Week of 05 July
11.	AstraZeneca / OXFORD	AZD1222	Australia TGA	Recombinant ChAdOx1 adenoviral vector encoding the Spike protein antigen of the SARS-CoV-2.	Submission from AZ received on 11 June	Several meetings held separately with AZ and TGA	✓	Ongoing	Anticipated date: Week of 05 July
12.	康希诺生物 CanSinoBIO	Ad5-nCoV	NMPA	Recombinant Novel Coronavirus Vaccine (Adenovirus Type 5 Vector)	✓	✓	Rolling data starting July 2021		
13.	Bharat Biotech, India	SARS-CoV-2 Vaccine, Inactivated (Vero Cell)/ COVAXIN	DCGI	**Whole-Virion Inactivated Vero Cell**	✓	✓	Rolling data starting July 2021		
14.	NOVAVAX	NVX-CoV2373/Covovax	EMA	Recombinant nanoparticle prefusion spike protein formulated with Matrix-M™ adjuvant.	✓	✓			
15.	Sinopharm / WIBP[2]	Inactivated SARS-CoV-2 Vaccine (Vero Cell)	NMPA	Inactivated, produced in Vero cells	✓	✓			
16.	CUREVAC the RNA people®	Zorecimeran (INN) concentrate and solvent for dispersion for injection; Company code: CVnCoV/CV07050101	EMA	mNRA-based vaccine encapsulated in lipid nanoparticle (LNP)	✓	Planned for 28 July 2021			
17.	Sanofi Pasteur	CoV2 preS dTM-AS03 vaccine	EMA	Recombinant, adjuvanted	✓	Planned for 9 July 2021			
18.	Vector State Research Centre of Viralogy and Biotechnology	EpiVacCorona	Russian NRA	Peptide antigen	Letter received not EOI. Reply sent on 15/01/2021				
19.	Zhifei Longcom, China	Recombinant Novel Coronavirus Vaccine（CHO Cell)	NMPA	Recombinant protein subunit	Response to 2nd EOI sent 29 Jan 2021. Additional information requested.				
20.	IMBCAMS, China	SARS-CoV-2 Vaccine, Inactivated (Vero Cell)	NMPA	Inactivated	Not accepted, still under initial development				
21.	Clover Biopharmaceuticals	SCB-2019	EMA	Novel recombinant SARS-CoV-2 Spike (S)-Trimer fusion protein	In discussion on submission strategy and timelines				

	Manufacturer / WHO EUL holder	Name of Vaccine	NRA of Record	Platform	EOI accepted	Pre-submission meeting held	Dossier accepted for review*	Status of assessment**	Decision date***
22.	BioCubaFarma - Cuba	Soberana 01,<br>Soberana 02<br>Soberana Plus<br>Abdala	CECMED	SARS-CoV-2 spike protein conjugated chemically to meningococcal B or tetanus toxoid or Aluminum	Awaiting information on strategy and timelines for submission.				

1. Beijing Institute of Biological Products Co-Ltd
2. Wuhan Institute of Biological Products Co Ltd

* Dossier Submission dates: more than one date is possible because of the rolling submission approach. Dossier is accepted after screening of received submission.
**Status of assessment: 1. Under screening; 2. Under assessment; 3. Waiting responses from the applicant. 4. Risk-benefit decision 5. Final decision made
*** Anticipated decision date: this is only an estimate because it depends on when all the data is submitted under rolling submission and when all the responses to the assessors' questions are submitted.

Please send any questions you may have to: WHOEUL@who.int
